# Identification of Serum Metabolites for Predicting Chronic Kidney Disease Progression according to Chronic Kidney Disease Cause

**DOI:** 10.3390/metabo12111125

**Published:** 2022-11-16

**Authors:** Eunjeong Kang, Yufei Li, Bora Kim, Ki Young Huh, Miyeun Han, Jung-Hyuck Ahn, Hye Youn Sung, Yong Seek Park, Seung Eun Lee, Sangjun Lee, Sue K. Park, Joo-Youn Cho, Kook-Hwan Oh

**Affiliations:** 1 Department of Internal Medicine, Ewha Womans University Seoul Hospital, Ewha Womans University College of Medicine, Seoul 07804, Republic of Korea; 2 Department of Clinical Pharmacology and Therapeutics, Seoul National University College of Medicine and Hospital, Seoul 03080, Republic of Korea; 3 Laboratory of Metabolism, Center for Cancer Research, National Cancer Institute, National Institutes of Health, Bethesda, MD 20892, USA; 4 Department of Internal Medicine, National Medical Center, Seoul 04564, Republic of Korea; 5 Department of Biochemistry, Ewha Womans University College of Medicine, Seoul 07804, Republic of Korea; 6 Department of Microbiology, School of Medicine, Kyung Hee University, Seoul 02447, Republic of Korea; 7 Department of Preventive Medicine, Seoul National University College of Medicine, Seoul 03080, Republic of Korea; 8 Cancer Research Institute, Seoul National University College of Medicine, Seoul 03080, Republic of Korea; 9 Department of Biomedical Sciences, Seoul National University Graduate School, Seoul 03080, Republic of Korea; 10 Integrated Major in Innovative Medical Science, Seoul National University College of Medicine, Seoul 03080, Republic of Korea; 11 Department of Internal Medicine, Seoul National University College of Medicine, Seoul 03080, Republic of Korea

**Keywords:** chronic kidney disease, disease progression, metabolomics, serum biomarkers

## Abstract

Early detection and proper management of chronic kidney disease (CKD) can delay progression to end-stage kidney disease. We applied metabolomics to discover novel biomarkers to predict the risk of deterioration in patients with different causes of CKD. We enrolled non-dialytic diabetic nephropathy (DMN, *n* = 124), hypertensive nephropathy (HTN, *n* = 118), and polycystic kidney disease (PKD, *n* = 124) patients from the KNOW-CKD cohort. Within each disease subgroup, subjects were categorized as progressors (P) or non-progressors (NP) based on the median eGFR slope. P and NP pairs were randomly selected after matching for age, sex, and baseline eGFR. Targeted metabolomics was performed to quantify 188 metabolites in the baseline serum samples. We selected ten progression-related biomarkers for DMN and nine biomarkers each for HTN and PKD. Clinical parameters showed good ability to predict DMN (AUC 0.734); however, this tendency was not evident for HTN (AUC 0.659) or PKD (AUC 0.560). Models constructed with selected metabolites and clinical parameters had better ability to predict CKD progression than clinical parameters only. When selected metabolites were used in combination with clinical indicators, random forest prediction models for CKD progression were constructed with AUCs of 0.826, 0.872, and 0.834 for DMN, HTN, and PKD, respectively. Select novel metabolites identified in this study can help identify high-risk CKD patients who may benefit from more aggressive medical treatment.

## 1. Introduction

Chronic kidney disease (CKD) is defined as an impairment in renal structure or function that has been present for more than 3 months [1]. The global prevalence of CKD is estimated to be 13.4% [2,3], which means that CKD is a global public health problem [4]. End-stage kidney disease (ESKD) refers to the stage of CKD where the kidneys can no longer function on their own, and dialysis or kidney transplant is required [5]. To reduce morbidities related to ESKD, Kidney Disease Improving Global Outcomes (KDIGO) guidelines recommend stratifying individuals at high risk of progression to ESKD to monitor them more closely [6]. Hence, to help improve patient management, more sensitive and early biomarkers of CKD progression are required.

Nonetheless, identifying progressive CKD patients is challenging due to the extensive variation in kidney function decline among individuals [7]. Current CKD blood indicators of kidney function, including serum creatinine and blood urea nitrogen, are insufficient to predict deterioration or provide insight into the underlying causative processes [8]. The kidneys are homeostatic regulators that function through filtration, reabsorption, secretion, synthesis, and degradation of various metabolites [9]. Therefore, metabolomics may identify biomarkers capable of predicting CKD progression. Previous studies have investigated cross-sectional associations between serum metabolite concentrations and renal function or CKD status [10,11]. However, it is not clear whether such associations are the result of reduced kidney function or whether changes in metabolite profiles are genuine causal drivers of CKD progression.

Additionally, although it is thought that there is a common pathophysiology underlying renal progression in CKD patients, the pace of CKD progression varies depending on the underlying etiology [12]. Diabetic nephropathy (DMN) and hypertensive nephropathy (HTN), which are complications of diabetes and chronic hypertension, respectively, are the two leading causes of CKD. Unlike DMN and HTN, polycystic kidney disease (PKD) is a genetic disorder that causes the uncontrolled growth of numerous cysts in the kidney [13]. These causes of CKD along with other characteristics, such as estimated glomerular filtration rate (eGFR), albuminuria category, and comorbid diseases, are considered significant predictors of outcomes in the KDIGO guidelines [6]. However, definitive markers to estimate the risk of deterioration of CKD to ESKD in diverse kidney diseases have not yet been identified. 

In the present study, we aimed to identify novel metabolic markers as indicators of disease progression among non-dialytic CKD patients according to the cause of CKD. In addition, we evaluated the ability of the novel metabolite markers to predict CKD progression as compared to clinical parameters. 

## 2. Materials and Methods

### 2.1. KNOW-CKD Cohort

The KNOW-CKD is an on-going multicenter prospective cohort study in Korea of 2238 patients aged between 20 and 75 years with non-dialyzed CKD stages 1–5 enrolled from February 2011 through January 2016. Detailed design and methods of the KNOW-CKD study have been described previously [14,15]. Based on the etiology of CKD, study subjects were categorized into the following subgroups of kidney diseases: glomerular disease, diabetic nephropathy (DMN), hypertensive nephropathy (HTN), and polycystic kidney disease (PKD) [14,15]. Subgroup classification was aided by pathologic diagnosis when kidney biopsy results were obtainable. If not, subgroup categorization was based on the clinical diagnosis of the nephrologist. Patients with type 2 diabetes who had albuminuria and diabetic retinopathy were classified as having DMN. Patients with hypertension and CKD in the absence of other diseases that could induce renal damage were identified as having HTN. Unified criteria were used to diagnose PKD [16]. For the current metabolomics study, only subjects in the DMN, HTN, and PKD subgroups were analyzed.

### 2.2. Ethical Approval 

The KNOW-CKD cohort study protocol was approved by the Institutional Review Board (IRB) of each participating clinical center—i.e., Seoul National University Hospital (1104-089-359), Seoul National University Bundang Hospital (B-1106/129-008), Yonsei University Severance Hospital (4-2011-0163), Kangbuk Samsung Medical Center (2011-01-076), Seoul St. Mary’s Hospital (KC11OIMI0441), Gil Hospital (GIRBA2553), Eulji General Hospital (201105-01), Chonnam National University Hospital (CNUH-2011-092), and Pusan Paik Hospital (11-091) in 2011. All procedures performed in this study involving human participants followed the ethical standards of the IRB at Seoul National University Hospital (IRB approval number 1710-032-891). Written informed consent was obtained from all the subjects when they were enrolled in KNOW-CKD cohort and all the samples processed in the lab are blinded and patients’ personal information were not disclosed. The study was conducted in accordance with the principles of the Declaration of Helsinki. 

### 2.3. Study Design 

Serum creatinine was measured for each subject in the KNOW-CKD study at every visit. Estimated GFR (eGFR) was calculated using the Chronic Kidney Disease Epidemiology Collaboration (CKD-EPI) equation [17]. In previous studies, GFR slope was found to be a strong surrogate end point of kidney disease progression that could replace end-stage kidney disease or doubling of serum creatinine in both early and advanced CKD [18]. For individual subjects, eGFR slope was calculated using a mixed model (random slope and random intercept method) based on repeatedly measured eGFR [19]. Since the decline of renal function over time is heavily dependent on the etiology of the kidney disease, we conducted metabolomics analyses separately within subgroups of CKD. Within each disease subgroup, subjects were categorized as progressors (P, case) or non-progressors (NP, control) based on the median eGFR slope for each subgroup. Within each subgroup, progressor-and-non-progressor pairs were selected randomly after best matching for age, sex, and baseline eGFR. Metabolomics assays were carried out on fasting serum samples obtained at study entry and preserved at −80 °C.

### 2.4. Targeted Metabolomics

Targeted metabolomics was performed using the AbsoluteIDQ^®^ p180 kit provided by Biocrates Life Science AG (Innsbruck, Austria). This commercial kit enables the quantitation of a total of 188 metabolites by multiple reaction monitoring in two analysis modes. In LC-MS/MS mode, 21 amino acids and 21 biogenic amines can be measured while in FIA-MS/MS mode, 1 hexose, 40 acylcarnitines, 90 glycerophospholipids, and 15 sphingolipids can be quantified. The LC-MS/MS system used in this study was an Agilent 1260 Infinity LC system (Santa Clara, CA, USA) equipped with an AB Sciex API 4000 QTRAP mass spectrometer (Foster City, CA, USA). A SecurityGuard C18 column (4 × 3 mm, No. AJO-4287) purchased from Phenomenex (Torrance, CA, USA) was connected to the LC system to separate analytes. Serum samples were prepared and analyzed according to the p180 kit manual. Internal standards, calibration standards, zero standards, and quality control samples are provided in the commercial kit. In brief, 10 μL of each serum sample was extracted and injected into the mass spectrometer for both LC-MS/MS analysis and FIA-MS/MS analysis. In LC mode, the mobile phase A contained 0.2% formic acid in water, while mobile phase B comprised 0.2% formic acid in ACN. Concentrations of each metabolite were calculated according to the corresponding calibration curves using Analyst^®^ 1.6.3 software (AB Sciex). Calibration curves and quality controls were evaluated using MetIDQ software (Biocrates Life Sciences AG). Data from different batches were normalized based on the same quality control samples in all batches. Metabolites with more than 50% outliers or missing values were removed before statistical analysis. 

### 2.5. Statistical Analysis

Baseline characteristics of enrolled participants are presented as mean ± standard deviation and median and interquartile range [IQR] for continuous variables and frequency (percentage) for categorical variables. To evaluate the significance of clinical differences between progressors and non-progressors, clinical indices were compared in each disease cohort by the Mann–Whitney U test. Univariate and multivariate analyses of targeted metabolites were conducted using MetaboAnalyst 5.0. Before analysis, Pareto scaling was carried out for data normalization. Wilcoxon rank-sum tests were performed to determine metabolites that were significantly different between progressors and non-progressors. To adjust the false discovery rate (FDR) in multiple comparison tests, Benjamini–Hochberg procedures were implemented. Among all the metabolites with a q value (FDR adjusted *p* value) less than 0.05, the ones with higher fold change between two progression status means were selected as biomarker candidates. For DMN and PKD, the fold change threshold was set to 1.2, while for the HTN group—in which more significant biomarkers were found than in the other two groups—the fold change cut-off was set to 1.5. Individual box-and-whisker plots of selected metabolites were drawn in Prism 9 software (version 9.4.1, GraphPad, CA, USA). To investigate the ability of the selected biomarkers to differentiate between disease progressors and non-progressors, principal component analysis (PCA) and heatmap analysis were performed. Euclidean distances and Ward’s linkage method were applied in the hierarchical clustering heatmap. 

### 2.6. Prediction Modeling and Network Analysis

We also used prediction modeling to analyze the relationship between the metabolite panels and disease progression outcomes. Prediction models were constructed to predict progression and non-progression outcomes using clinical indices alone or with the selected metabolites. Six of the clinical parameters most commonly used to evaluate progression (age, sex, mean arterial pressure (MAP), baseline eGFR, body mass index [BMI], and random urine protein/creatinine ratio [uPCR]) were selected by prediction modeling. For each disease group, logistic regression models were constructed using clinical parameters only and clinical parameters in conjunction with the selected progression-related biomarkers. The random forest (RF) algorithm, which is a reliable classification tool that can achieve a high level of accuracy in predicting outcomes, was adopted in this study. To validate each model across different combinations of the datasets, 5-fold cross-validation was adopted and the mean AUC was calculated. Receiver operating characteristic (ROC) curves of the three models were generated using the pROC package in R (version 4.1.3). The significance of differences in AUC values between pairwise models was evaluated by DeLong’s test. To map the biochemical relationships among all statistically significant metabolites, MetaMapp network analysis was performed for each CKD disease group. Information including PubChem compound identifier number (CID), Kyoto Encyclopedia of Genes and Genomes identifier number (KEGG ID), q value, metabolite fold change, and simplified molecular-input line-entry system (SMILES) string of each metabolite was organized before analysis. Global interaction network files were generated based on KEGG and PubChem databases and converted into Cytoscape SIF files by MetaMapp in Google Colaboratory (Colab). Cytoscape (version 3.9.1) was used to visualize metabolic networks, including generated nodes and edge attributes. 

## 3. Results

### 3.1. Baseline Characteristics of the Study Participants

**Figure 1:**
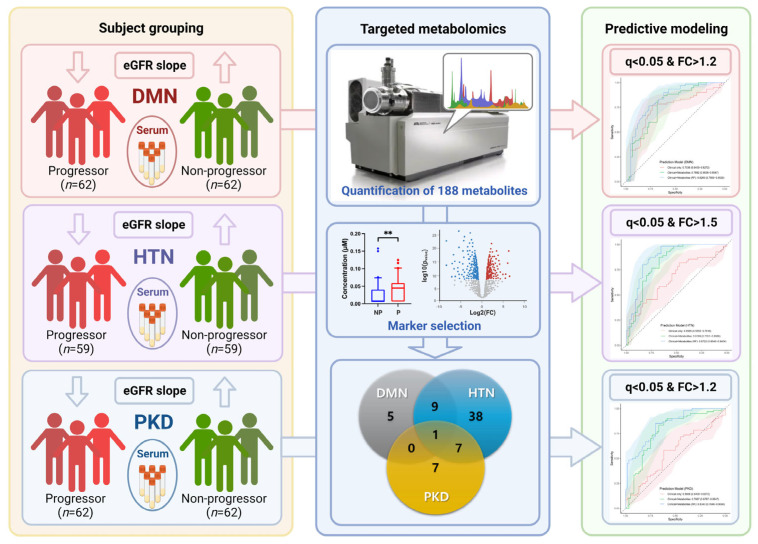
Schematic description of this study. Clinical data and serum samples from three chronic kidney disease cohorts, namely diabetic nephropathy (DMN, *n* = 124), hypertensive nephropathy (HTN, *n* = 118), and polycystic kidney disease (PKD, *n* = 124), were collected. In each disease group, half of the patients with lower eGFR slope were defined as disease-progressors, while the others were defined as non-progressors. Other clinical parameters including age, sex, and baseline eGFR, were confirmed to have no significant differences between progressors and non-progressors. Targeted metabolomics was performed to quantify 188 metabolites in the baseline serum samples of all the cohorts. For each group, disease progression-related metabolite biomarkers were selected based on statistical analysis. Finally, predictive models were generated and validated through 5-fold cross-validation. Images were created with BioRender.com.

Overall workflow and study design are presented in Figure 1. Three disease cohorts were evaluated in this study: DMN, HTN, and PKD. Baseline characteristics of the KNOW-CKD study sample according to the etiology of CKD are shown in Table 1. There were no significant differences in sex, mean age, or mean baseline eGFR between progressors and non-progressors within the three disease groups. 

### 3.2. Potential Metabolic Biomarkers of Diabetic Nephropathy (DMN)

**Figure 2:**
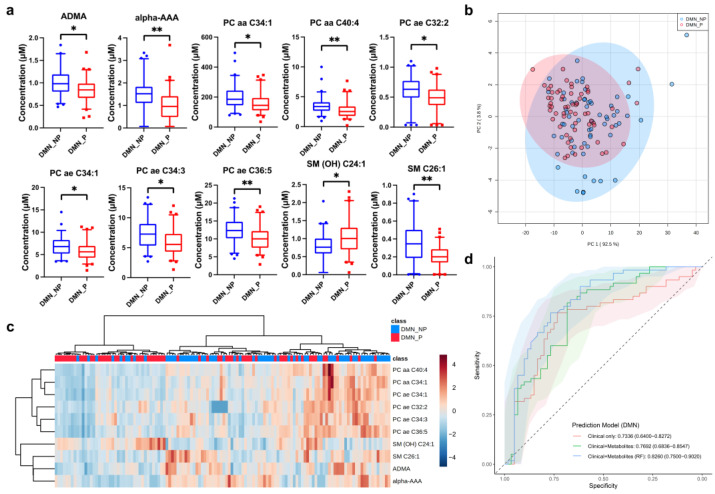
Multivariate statistical analysis and predictive modeling of the selected DMN progression-related biomarkers. (**a**) Individual box-and-whisker plot (median, 5–95 percentile) of the selected metabolites in DMN progressors (P, red) and non-progressors (NP, blue). (**b**) PCA of two components of selected metabolites from DMN progressors (P, red) and non-progressors (NP, blue). (**c**) Heatmap and hierarchical clustering analysis of the selected metabolites from DMN progressors (P, red) and non-progressors (NP, blue). (**d**). ROC curves for three prediction models of DMN progression: models using clinical parameters only (red) or together with selected metabolite biomarkers (green: by logistic regression; blue: by random forest). Solid line: mean AUC of the 5-fold cross-validation. Shaded area: 95% CI of AUC. * q < 0.05, ** q < 0.01. PC aa: diacyl-phosphatidylcholine; PC ae: acyl-alkyl-phosphatidylcholine.

After removing unreliable quantitation results, we were left with results for 137 serum metabolites for DMN patients. Among these metabolites, only 15 metabolites showed significant differences between DMN progressors and non-progressors (q < 0.05, Table S1). Most of these metabolites showed decreased abundance in progressive subjects. To reduce the number of biomarkers for effective modeling, ten metabolites that had a fold-change threshold of over 1.2 were retained, namely asymmetric dimethylarginine (ADMA), L-2-aminoadipic acid (alpha-AAA), PC aa C34:1, PC aa C40:4, PC ae C32:2, PC ae C34:1, PC ae C34:3, PC ae C36:5, SM (OH) C24:1, SM C26:1 (Table 2, Figure 2a). 

To evaluate the ability of these 10 metabolic biomarkers to distinguish progressors and non-progressors, unsupervised multivariate analyses were performed. Although obvious separation of the two groups was not observed in the PCA plot, the distribution trends of the two groups were different (Figure 2b). In the cluster heatmap, progressors and non-progressors did not cluster separately (Figure 2c). The logistic regression model based on clinical parameters only achieved a decent prediction ability with a mean AUC of 0.734. Addition of metabolite data increased the average AUC of the logistic regression model slightly to 0.769 but without statistical significance (Table S2). However, when the RF algorithm was used, the mean AUC of the prediction model containing both clinical parameters and biomarker data improved to 0.826 (*p* < 0.05, Figure 2d, Table 3).

### 3.3. Potential Metabolic Biomarkers for Hypertensive Nephropathy (HTN)

**Figure 3:**
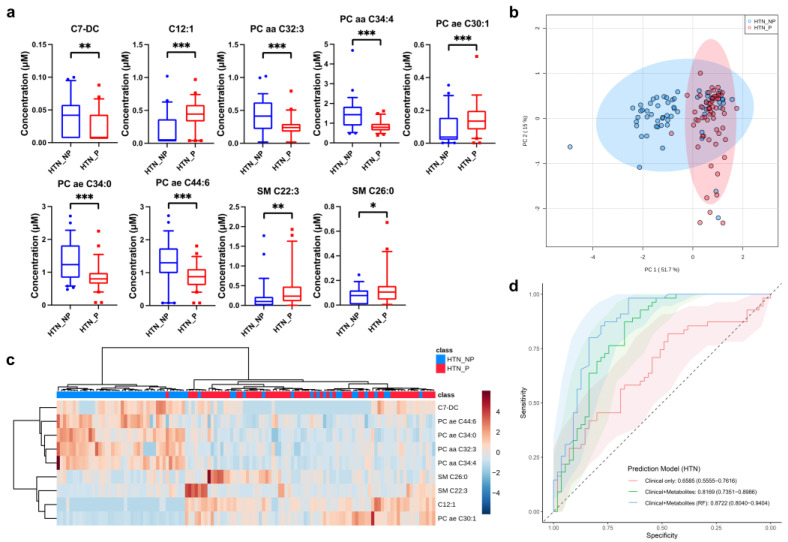
Multivariate statistical analysis and predictive modeling of the selected HTN progression-related biomarkers. (**a**) Individual box-and-whisker plot (median, 5–95 percentile) of the selected metabolites in HTN progressors (P, red) and non-progressors (NP, blue). (**b**) PCA of two components of selected metabolites from HTN progressors (P, red) and non-progressors (NP, blue). (**c**) Heatmap and hierarchical clustering analysis of the selected metabolites from HTN progressors (P, red) and non-progressors (NP, blue). (**d**) ROC curves for three prediction models of HTN progression: models using clinical parameters only (red) or together with selected metabolite biomarkers (green: by logistic regression; blue: by random forest). Solid line: mean AUC of the 5-fold cross-validation. Shaded area: 95% CI of AUC. * q < 0.05, ** q < 0.01, *** q < 0.001. PC aa: diacyl-phosphatidylcholine; PC ae: acyl-alkyl-phosphatidylcholine.

For the HTN cohort, 139 serum metabolites were measured successfully. Fifty-five metabolic biomarker candidates were found to differentiate HTN progressors and non-progressors (q < 0.05, Table S1). Most of the biomarker candidates decreased in concentration in HTN progressors. To find biomarkers representative of HTN disease progression, the fold-change threshold was set to 1.5. Ultimately, nine metabolites were selected as HTN progressive biomarkers, namely dodecenoylcarnitine (C12:1), pimelylcarnitine (C7-DC), PC aa C32:3, PC aa C34:4, PC ae C30:1, PC ae C34:0, PC ae C44:6, SM C22:3, SM C26:0 (Table 2, Figure 3a). 

According to the PCA plot based on this metabolite panel, even though there was overlap between progressors and non-progressors, there were obvious differences in sample distributions and directionality (Figure 3b). Moreover, the heatmap showed distinct differences between the two disease status cohorts for the nine selected biomarkers, and hierarchical clustering distinguished between these two groups well (Figure 3c). Clinical indicators appeared to be somewhat underpowered in predicting disease progression by logistic regression models, with a mean AUC of 0.659. The combination of clinical parameters and metabolite data resulted in a significant improvement in the logistic regression model, as demonstrated by a high average AUC of 0.817 (*p* < 0.005). Moreover, the predictive ability of the combined biomarkers was enhanced significantly in the RF model, with a 5-fold AUC ranging from 0.804 to 0.940 (*p* < 0.05, Figure 3d, Table 3).

### 3.4. Potential Metabolic Biomarkers for Polycystic Kidney Disease (PKD)

**Figure 4:**
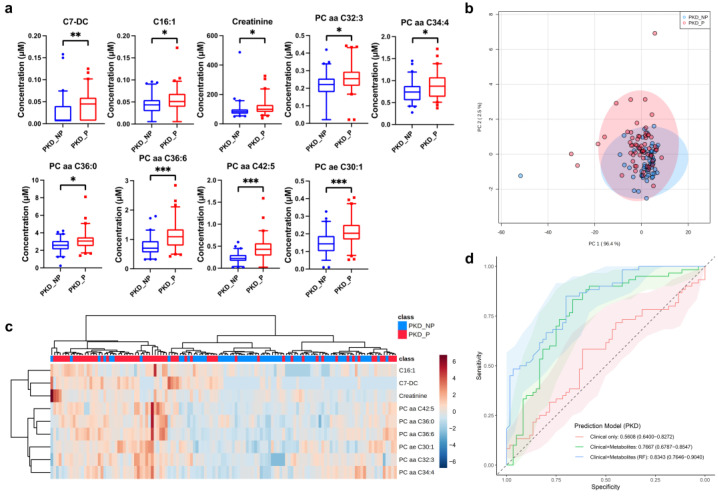
Multivariate statistical analysis and predictive modeling of the selected PKD progression-related biomarkers. (**a**) Individual box-and-whisker plot (median, 5–95 percentile) of the selected metabolites in PKD progressors (P, red) and non-progressors (NP, blue). (**b**) PCA of two components of selected metabolites from PKD progressors (P, red) and non-progressors (NP, blue). (**c**) Heatmap and hierarchical clustering analysis of the selected metabolites from PKD progressors (P, red) and non-progressors (NP, blue). (**d**) ROC curves for three prediction models of PKD progression: models using clinical parameters only (red) or together with selected metabolite biomarkers (green: by logistic regression; blue: by random forest). Solid line: mean AUC of the 5-fold cross-validation. Shaded area: 95% CI of AUC. * q < 0.05, ** q < 0.01, *** q < 0.001. PC aa: diacyl-phosphatidylcholine; PC ae: acyl-alkyl-phosphatidylcholine.

One hundred and thirty-four metabolites were validated in serum metabolite quantitation of PKD cohorts. Fifteen of the metabolites with a q value less than 0.05 were selected as potential metabolic biomarker candidates (Table S1). Unlike the other two disease cohorts, almost all of these biomarker candidates tended to have increased concentrations in the PKD progressive cohort. Metabolites with a fold-change of less than 1.2 were removed from the biomarker candidates to construct a more efficient predictive model. Eventually, nine metabolites, namely hexadecenoylcarnitine (C16:1), C7-DC, creatinine, PC aa C32:3, PC aa C34:4, PC aa C36:0, PC aa C36:6, PC aa C42:5, PC ae C30:1 were chosen as PKD progression-related biomarkers (Table 2, Figure 4a).

PKD non-progressors were not well separated from progressors based on the 9-metabolite panel when using unsupervised PCA (Figure 4b). In the cluster heatmap, progressors and non-progressors did not cluster completely separately, but local aggregations of members of these two groups were evident (Figure 4c). Clinical indices did not classify PKD progressors and non-progressors well, with a low mean AUC of 0.5608. However, when metabolite results were added to the clinical data for classification, the predictive ability of the logistic regression model improved considerably (mean AUC of 0.767, *p* < 0.001). Similar to the other two groups, the RF model based on clinical and metabolite data had the best predictive performance with an average AUC of 0.834 (*p* < 0.05, Figure 4d, Table 3).

### 3.5. Metabolite Network Analysis

**Figure 5:**
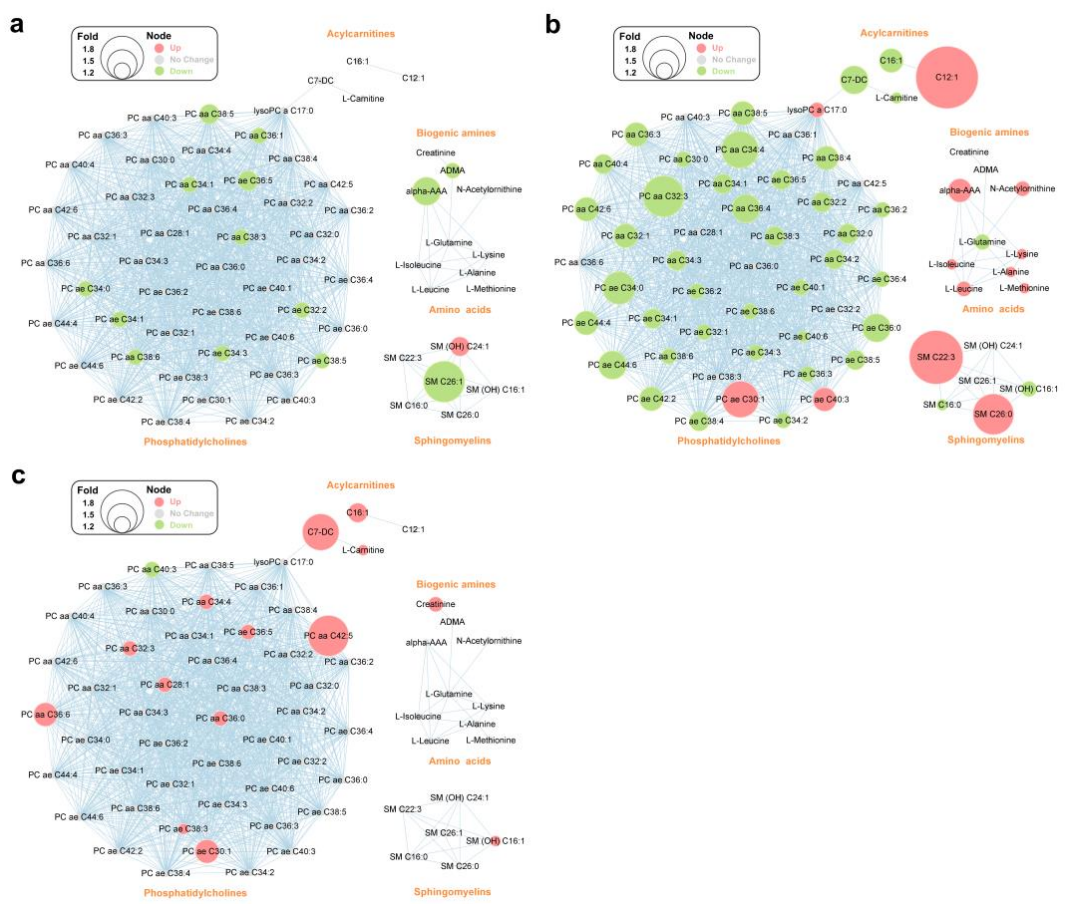
Changes in metabolic patterns of disease progressors compared to non-progressors in the three disease groups. MetaMapp metabolite networks of 67 metabolites altered in DMN (**a**), HTN (**b**), and PKD (**c**), with nodes representing metabolites and edges, indicating chemical relationships. ADMA, asymmetric dimethylarginine; alpha-AAA, L-2-aminoadipic acid; C7-DC, pimelylcarnitine; C12:1, dodecenoylcarnitine; C16:1, hexadecenoylcarnitine; PC aa: diacyl-phosphatidylcholine; PC ae: acyl-alkyl-phosphatidylcholine.

To interpret changes in the metabolic patterns in the three disease groups, all significant metabolites (q < 0.05) in the DMN, HTN, and PKD subgroups were analyzed using MetaMapp (Table S1). Accordingly, biochemical interactions of a total of 67 metabolites were mapped based on their chemical structures and functional groups. Metabolic biomarkers in the HTN group showed the most significant differences between progressors and non-progressors (Figure 5). Biogenic amines like ADMA and alpha-AAA decreased only in progressive DMN, and phosphatidylcholines including both diacyl-phosphatidylcholines (PC aa) and acyl-alkyl-phosphatidylcholines (PC ae) showed a slight downward trend in DMN progressors (Figure 5a). Similarly, PCs except for one lyso PC and two PC ae decreased dramatically in HTN progressors (Figure 5b). Unlike the other two groups, amino acids and biogenic amines related to glutamine metabolism tended to be higher in HTN progressors. One acylcarnitine and two sphingomyelins showed a considerable increase in HTN progressors. Interestingly, metabolic biomarkers like PC aa and acylcarnitines tended to be higher in PKD progressors, which was the complete opposite of what was observed in the other two disease groups (Figure 5c).

## 4. Discussion

CKD is usually classified into five stages based on biomarkers such as serum creatinine and proteinuria [20]. Due to the heterogeneity in kidney function decline among individuals, evaluating the progression of CKD to ESKD in patients based on CKD stage is challenging [21]. Therefore, we applied metabolomics to discover serum biomarkers that can better predict the risk of progression of three different types of CKD—namely DMN, HTN, and PKD—than existing biomarkers. For all three groups, selected metabolite biomarkers were used to construct CKD progression diagnostic models. In HTN and PKD disease group, the predictive models based on the combination of selected novel metabolomics biomarkers and clinical parameters were better able to predict disease progression than the models based on clinical parameters alone.

An important strength of this study is that we successfully constructed predictive models for three different disease groups. Although clinical indices such as eGFR, proteinuria, and mean blood pressure have been validated for CKD classification [22], they are often not effective at identifying high-risk subjects among patients with similar clinical symptoms. In our study, clinical indicators only showed good predictive power in the DMN group. The combination of metabolomics results and clinical data addressed this issue. In the HTN and PKD groups, even though the clinical classifiers showed much lower predictive ability than in the DMN group, the addition of metabolomics data improved the classification power of these models to a greater extent than observed in the DMN group. When a logistic regression method was adopted, the presence or absence of metabolite data did not significantly affect the performance of the model to predict disease progression in DMN. However, the selected biomarkers showed high predictive power when modeled using a machine learning approach. Random forest models exhibited better ability to predict disease deterioration than logistic regression models in all three disease groups. This highlights the potential of using machine learning methods in clinical medicine.

The causes of CKD are varied. DMN is one of the most profound consequences of diabetes mellitus in the general adult population of the US [23,24] and has become the leading cause of ESKD in Korea [25] and across the world. Hypertension, which can cause damage to the blood vessels and filters in the kidney, is also one of the main risk factors for the development and progression of CKD [26,27]. These two diseases are caused by a variety of factors, including environmental or secondary effects of other chronic diseases.

One of our findings is that a prediction model based on clinical indicators is sufficient to predict DMN deterioration without the addition of metabolic biomarkers. Several predictive models for CKD progression in diabetic patients have been developed [28,29,30,31]. Most previous studies used clinical parameters while others included laboratory findings such as age, eGFR, albuminuria, hemoglobin A1c, diabetic retinopathy, and diabetes duration. The AUC values of these prediction models were usually more than 0.7, including in the current study (Figure 2d), indicating fair performance in a clinical setting. Since the addition of metabolites to the predictive model did not improve the prediction ability of the model, it can be inferred that DMN progression reflects clinical phenotype more than it does changes in the metabolomic phenotype. Although microalbuminuria is generally thought to be the earliest marker of DMN and is an important predictor of CKD progression in clinical practice, more than 30% of patients with type 2 diabetes may have renal function decline before microalbuminuria [32,33,34]. Novel biomarkers to recognize DMN without albuminuria to improve clinical outcomes are required, and metabolomics has the potential to identify these markers. In this study, because many DMN patients already had albuminuria or proteinuria and subjects with advanced stage CKD were included, additional studies in different cohorts are needed to identify novel metabolites that can identify non-albuminuria DMN patients.

HTN, represented by hypertensive nephrosclerosis, is less often diagnosed with kidney biopsy than other causative diseases of CKD, and its clinical phenotypes are diverse, so the diagnostic criteria for this disease entity remain controversial [35,36,37,38]. Therefore, few studies have attempted to predict CKD progression in those with CKD caused by HTN. Clinical indicators such as long-standing hypertension, no diabetes, no hematuria, and no overt proteinuria have shown a positive predictive value of 97% in African Americans and 4% in Italians [38,39]. Thus, it is difficult to diagnose and predict HTN with clinical parameters alone, and novel biomarkers to predict CKD progression in this group are essential. Although additional validation studies are needed, the combination of clinical indicators and metabolites identified in this study significantly improved the ability of our model to predict CKD progression in the HTN group (Figure 3d).

Metabolic biomarkers differentiating between HTN progressors and non-progressors showed the most significant differences and the highest ability to predict disease progression. Phosphatidylcholines and sphingomyelins, which are mediators linking lipid-induced inflammatory pathways [40] and have been shown to be associated with renal impairment [41], showed the most significant changes between progressors and non-progressors in the HTN subgroup. Sphingomyelin, a type of sphingolipid found in cellular membranes, has been reported as a significant biochemical covariate of urinary albumin excretion in renal disease [42]. Sphingomyelin levels in the plasma of hypertensive rats were found to be increased, which was ameliorated by administration of a cardio-renal protective drug [43]. Amino acids as the building blocks of proteins play a vital role in metabolic processes and organ function [44]. Elevated levels of plasma amino acids have been shown to correlate with a reduction in kidney function [45]. Kidneys take up glutamine from arterial blood and release a small amount of lysine, leucine, and isoleucine into the systemic circulation [46]. In our study, amino acids except for glutamine were significantly higher in HTN progressors than non-progressors, indicating a decline in kidney function in progressive HTN. In addition, the observed upward trends of alpha-AAA and N-acetylornithine in HTN progressors also suggest that deficiencies in renal energy sources like glutamine and accumulation of amino acid are biomarkers of HTN progression.

PKD is the most prevalent inherited kidney disease and is characterized by gradual enlargement of multiple cysts in the kidneys along with loss of renal function over decades [13]. The distinct metabolic patterns found in progressive PKD probably compared with DMN and HTN are likely due to different underlying pathologies. Our data confirmed this; phosphatidylcholines and acylcarnitines decreased significantly in the DMN and HTN groups but showed an increasing trend in PKD subjects. One of the most important indicators of renal function decline in PKD patients is mutation of the PKD1 and PKD2 genes. Patients with PKD1 mutations present with more aggressive kidney disease than those with PKD2 mutations, so renal replacement therapy is performed at a younger age in patients with PKD1 gene mutations [47,48,49]. Another key factor that can reflect the deterioration of renal function in PKD patients is height-adjusted total kidney volume (htTKV). In the Consortium for Radiologic Imaging observational Study (CRISP study), a baseline htTKV of 600 cc/m predicted the risk of reaching CKD stage 3 within 8 years [50]. Pathophysiological similarities between PKD and malignancy—both of which are related to rapid abnormal cell proliferation [51]—have been reported, and it has been suggested that there may be a common pathway between PKD and cancer [52]. Therefore, different metabolite patterns in PKD patients are observed not only because of common mechanisms of CKD progression shared with DMN and HTN, but also genetic factors and rapidly increasing kidney volumes as a result of excessive cell proliferation.

Our study had several limitations. Firstly, we were not able to validate our findings in a replication cohort due to the limited sample size. It may result in worse performance and credibility in our diagnostic models. To avoid this problem, we introduced a 5-fold cross-validation method into our diagnosis models for internal validation. The multiple random train-test splits make it possible to control the randomness for the reproducibility of our results. Secondly, proteinuria levels of CKD progressors and non-progressors, which is one of the important risk factors for CKD progression, were not matched in our study. This may be the reason why the clinical indicators in the DMN group showed good predictive power on CKD progression. Lastly, since the KNOW-CKD cohort recruited clinical data and samples from Korean patients, the results of this study may be not applicable to people in other regions. Although results from a larger population from diverse regions are better for obtaining accurate and usually applicable results, we still consider our study valuable because it can provide ideas and clues for follow-up research.

## 5. Conclusions

In conclusion, the novel biomarkers described in this study can help identify high-risk CKD patients who may benefit from more aggressive medical treatment and help to distinguish therapy responders from non-responders for more effective patient management.

## Figures and Tables

**Table 1:** Baseline characteristics of enrolled patients according to the cause of chronic kidney disease.

Group	Characteristic	Non-Progressor	Progressor	*p*
DMN	Subject	62 (50%)	62 (50%)	0.999
	Male sex	43 (69.4%)	43 (69.4%)	0.999
	Age (years)	61.0 [51.0; 65.0]	60.0 [53.0; 64.0]	0.622
	Baseline eGFR (mL/min/1.73 m^2^)	41.8 [35.2; 49.6]	42.2 [34.7; 49.9]	0.871
	eGFR slope (mL/min/1.73 m^2^/year)	0.2 ± 1.3	−3.2 ± 1.3	<0.001
	Systolic BP (mmHg)	129.3 ± 16.3	133.0 ± 16.8	0.207
	Diastolic BP (mmHg)	75.1 ± 9.5	75.1 ± 9.8	0.985
	BMI (kg/m^2^)	24.8 [23.0; 27.1]	24.8 [23.1; 26.6]	0.928
	uPCR (g/g)	0.38 [0.23; 0.76]	2.17 [1.04; 4.28]	<0.001
HTN	Subject	59 (50%)	59 (50%)	0.999
	Male	46 (77.97%)	37 (62.7%)	0.107
	Age (years)	62.00 [55.0; 67.5]	60.0 [56.0; 68.0]	0.948
	Baseline eGFR (mL/min/1.73 m^2^)	33.30 [24.4; 45.0]	30.4 [25.9; 41.2]	0.823
	eGFR slope (mL/min/1.73 m^2^/year)	0.6 [0.1; 1.3]	−1.5 [−2.7; −1.2]	<0.001
	Systolic BP (mmHg)	122.7 ± 13.8	124.1 ± 14.8	0.603
	Diastolic BP (mmHg)	75.1 ± 10.9	74.9 ± 9.8	0.951
	BMI (kg/m^2^)	25.2 ± 3.4	24.9 ± 3.2	0.684
	uPCR (g/g)	0.1 [0.05; 0.4]	0.6 [0.2; 1.1]	<0.001
PKD	Subject	62 (50%)	62 (50%)	0.999
	Male	40 (64.52%)	40 (64.5%)	0.999
	Age (years)	46.5 ± 11.1	45.7 ± 8.6	0.679
	Baseline eGFR (mL/min/1.73 m^2^)	81.3 [63.5; 106.6]	71.3 [60.9; 100.0]	0.202
	eGFR slope (mL/min/1.73 m^2^/year)	0.4 [−0.3; 1.2]	−2.8 [−4.2; −1.7]	<0.001
	Systolic BP (mmHg)	127.6 ± 12.6	129.9 ± 11.8	0.302
	Diastolic BP (mmHg)	80.7 ± 9.3	81.5 ± 9.9	0.615
	BMI (kg/m^2^)	23.2 [21.7; 25.2]	23.1 [21.6; 25.8]	0.656
	uPCR (g/g)	0.06 [0.04; 0.15]	0.1 [ 0.0; 0.3]	0.020

DMN, diabetic nephropathy; HTN, hypertensive nephropathy; PKD, polycystic kidney disease; eGFR, estimated glomerular filtration rate; BP, blood pressure; BMI, body mass index; uPCR, urine protein-to-creatinine ratio.

**Table 2:** List of the selected disease progression related biomarkers in each disease group.

Group	Metabolite	Category	q	FC
DMN	SM C26:1	Sphingomyelin	0.002	0.567
	L-2-Aminoadipic acid (alpha-AAA)	Biogenic amine	0.003	0.666
	PC ae C36:5	Phosphatidylcholine	0.004	0.805
	PC aa C40:4	Phosphatidylcholine	0.008	0.785
	PC aa C34:1	Phosphatidylcholine	0.019	0.790
	Asymmetric dimethylarginine (ADMA)	Biogenic amine	0.019	0.808
	PC ae C34:1	Phosphatidylcholine	0.028	0.830
	SM (OH) C24:1	Sphingomyelin	0.028	1.323
	PC ae C34:3	Phosphatidylcholine	0.029	0.816
	PC ae C32:2	Phosphatidylcholine	0.030	0.821
HTN	Dodecenoylcarnitine (C12:1)	Acylcarnitine	<0.001	2.257
	PC aa C34:4	Phosphatidylcholine	<0.001	0.580
	PC ae C34:0	Phosphatidylcholine	<0.001	0.631
	PC ae C44:6	Phosphatidylcholine	<0.001	0.653
	PC aa C32:3	Phosphatidylcholine	<0.001	0.565
	PC ae C30:1	Phosphatidylcholine	<0.001	1.726
	SM C22:3	Sphingomyelin	0.007	2.085
	Pimelylcarnitine (C7-DC)	Acylcarnitine	0.010	0.658
	SM C26:0	Sphingomyelin	0.034	1.834
PKD	PC aa C42:5	Phosphatidylcholine	<0.001	1.846
	PC aa C36:6	Phosphatidylcholine	<0.001	1.449
	PC ae C30:1	Phosphatidylcholine	<0.001	1.407
	Pimelylcarnitine (C7-DC)	Acylcarnitine	0.007	1.666
	PC aa C32:3	Phosphatidylcholine	0.010	1.227
	PC aa C36:0	Phosphatidylcholine	0.013	1.225
	Creatinine	Biogenic amine	0.015	1.200
	PC aa C34:4	Phosphatidylcholine	0.022	1.201
	Hexadecenoylcarnitine (C16:1)	Acylcarnitine	0.025	1.316

q: FDR-adjusted *p* value; FC: fold change of the metabolites in progressor with respect to non-progressor; DMN, diabetic nephropathy; HTN, hypertensive nephropathy; PKD, polycystic kidney disease; PC aa: diacyl-phosphatidylcholine; PC ae: acyl-alkyl-phosphatidylcholine.

**Table 3:** Classification performance metrics for each predictive model.

Group	Model	Mean AUC	Accuracy	Sensitivity	Specificity	F1 Score
DMN	Model 1	0.734	0.71	0.80	0.62	0.73
	Model 2	0.770	0.72	0.68	0.75	0.71
	Model 3	0.826	0.74	0.67	0.82	0.71
HTN	Model 1	0.659	0.62	0.65	0.58	0.63
	Model 2	0.817	0.75	0.67	0.82	0.72
	Model 3	0.872	0.79	0.71	0.87	0.77
PKD	Model 1	0.561	0.58	0.58	0.58	0.58
	Model 2	0.767	0.72	0.72	0.73	0.72
	Model 3	0.834	0.72	0.70	0.73	0.71

Model 1, clinical only; Model 2, clinical + metabolites; Model 3, clinical + metabolites in random forest.

## Data Availability

The data that support the findings of this study are available on reasonable request. The request can initially be sent to the corresponding author, then will be distributed to the investigators. The relevant data, analytical methods, and study materials will be open to researchers after a comprehensive discussion.

## Supplementary Materials

The following supporting information can be downloaded at: https://www.mdpi.com/article/10.3390/metabo12111125/s1, Table S1. List of all significantly changed metabolites between progressors and non-progressors; Table S2. DeLong’s test for pairwise correlated ROC curves.
