# Supplementary material for: Identification of Serum Metabolites for Predicting Chronic Kidney Disease Progression according to Chronic Kidney Disease Cause
Source: Metabolites. 2022 Nov 16;12(11):1125. doi: 10.3390/metabo12111125 (PMC9696352; doi:10.3390/metabo12111125)
Supplement: Supplementary file 1 [file metabolites-12-01125-s001.zip › metabolites-1990174-supplementary.pdf]

Supplementary materials

Table S1. List of all significantly changed metabolites between progressors and non-progressors.

Group	Metabolite	Category	*q*	FC
DMN	SM C26:1	Sphingomyelin	0.0021	0.567
	L-2-Aminoadipic acid (alpha-AAA)	Biogenic amine	0.0034	0.666
	PC ae C36:5	Phosphatidylcholine	0.0037	0.805
	PC aa C40:4	Phosphatidylcholine	0.0079	0.785
	PC aa C34:1	Phosphatidylcholine	0.0185	0.790
	PC ae C38:5	Phosphatidylcholine	0.0185	0.848
	Asymmetric dimethylarginine (ADMA)	Biogenic amine	0.0192	0.808
	PC ae C36:4	Phosphatidylcholine	0.0266	0.855
	PC ae C34:1	Phosphatidylcholine	0.0283	0.830
	SM (OH) C24:1	Sphingomyelin	0.0283	1.323
	PC ae C34:3	Phosphatidylcholine	0.0294	0.816
	PC ae C32:2	Phosphatidylcholine	0.0305	0.821
	PC aa C38:3	Phosphatidylcholine	0.0377	0.844
	PC aa C36:1	Phosphatidylcholine	0.0377	0.848
	PC ae C34:0	Phosphatidylcholine	0.0498	0.833
HTN	Dodecenoylcarnitine (C12:1)	Acylcarnitine	1.04E-06	2.257
	PC aa C36:4	Phosphatidylcholine	1.15E-06	0.687
	PC aa C34:2	Phosphatidylcholine	2.09E-06	0.758
	PC aa C34:4	Phosphatidylcholine	2.39E-06	0.580
	PC ae C34:0	Phosphatidylcholine	1.18E-05	0.631
	PC ae C44:6	Phosphatidylcholine	3.59E-05	0.653
	PC aa C36:3	Phosphatidylcholine	3.59E-05	0.740
	PC aa C38:4	Phosphatidylcholine	4.68E-05	0.708
	PC aa C36:2	Phosphatidylcholine	9.65E-05	0.828
	PC aa C32:3	Phosphatidylcholine	1.32E-04	0.565
	PC ae C38:5	Phosphatidylcholine	1.32E-04	0.796
	L-Glutamine	Amino acid	2.20E-04	0.864
	PC ae C34:1	Phosphatidylcholine	2.40E-04	0.782
	PC ae C36:5	Phosphatidylcholine	2.41E-04	0.754

PC aa C34:1	Phosphatidylcholine	2.41E-04	0.775
PC ae C30:1	Phosphatidylcholine	2.43E-04	1.726
PC aa C38:3	Phosphatidylcholine	2.43E-04	0.756
PC ae C32:1	Phosphatidylcholine	2.44E-04	0.812
PC aa C32:0	Phosphatidylcholine	2.44E-04	0.775
SM (OH) C16:1	Sphingomyelin	3.97E-04	0.803
PC ae C36:2	Phosphatidylcholine	9.49E-04	0.819
PC ae C38:4	Phosphatidylcholine	9.49E-04	0.792
PC ae C40:3	Phosphatidylcholine	0.0012	1.430
Hexadecenoylcarnitine (C16:1)	Acylcarnitine	0.0020	0.724
PC ae C36:3	Phosphatidylcholine	0.0020	0.822
PC ae C36:4	Phosphatidylcholine	0.0021	0.806
PC aa C38:6	Phosphatidylcholine	0.0021	0.804
PC ae C42:2	Phosphatidylcholine	0.0024	0.738
PC aa C34:3	Phosphatidylcholine	0.0025	0.749
PC aa C38:5	Phosphatidylcholine	0.0030	0.768
PC aa C40:4	Phosphatidylcholine	0.0030	0.715
PC ae C34:2	Phosphatidylcholine	0.0031	0.838
PC ae C34:3	Phosphatidylcholine	0.0046	0.808
PC aa C42:6	Phosphatidylcholine	0.0049	0.736
SM C22:3	Sphingomyelin	0.0068	2.085
PC aa C32:2	Phosphatidylcholine	0.0072	0.747
L-Leucine	Amino acid	0.0076	1.151
Pimelylcarnitine (C7-DC)	Acylcarnitine	0.0100	0.658
PC ae C36:0	Phosphatidylcholine	0.0111	0.667
PC ae C38:6	Phosphatidylcholine	0.0113	0.846
PC ae C40:1	Phosphatidylcholine	0.0148	0.803
PC aa C30:0	Phosphatidylcholine	0.0160	0.819
PC ae C44:4	Phosphatidylcholine	0.0226	0.709
SM C16:0	Sphingomyelin	0.0241	0.904
alpha-AAA	Biogenic amine	0.0267	1.411
L-Isoleucine	Amino acid	0.0275	1.101
L-Alanine	Amino acid	0.0284	1.097
N-Acetylornithine	Biogenic amine	0.0284	1.242
lysoPC a C17:0	Phosphatidylcholine	0.0287	1.196

	SM C26:0	Sphingomyelin	0.0340	1.834
	L-Carnitine	Acylcarnitine	0.0369	0.880
	PC ae C40:6	Phosphatidylcholine	0.0382	0.893
	L-Lysine	Amino acid	0.0393	1.070
	PC aa C32:1	Phosphatidylcholine	0.0395	0.717
	L-Methionine	Amino acid	0.0417	1.095
**PKD**	PC aa C42:5	Phosphatidylcholine	7.90E-06	1.846
	PC aa C36:6	Phosphatidylcholine	5.31E-05	1.449
	PC ae C30:1	Phosphatidylcholine	7.37E-04	1.407
	PC aa C28:1	Phosphatidylcholine	0.0022	1.185
	Pimelylcarnitine (C7-DC)	Acylcarnitine	0.0075	1.666
	PC aa C34:1	Phosphatidylcholine	0.0101	1.152
	PC aa C32:3	Phosphatidylcholine	0.0101	1.227
	PC aa C36:0	Phosphatidylcholine	0.0128	1.225
	Creatinine	Biogenic amine	0.0150	1.200
	PC aa C40:3	Phosphatidylcholine	0.0150	0.840
	L-Carnitine	Acylcarnitine	0.0219	1.135
	PC aa C34:4	Phosphatidylcholine	0.0219	1.201
	SM (OH) C16:1	Sphingomyelin	0.0233	1.139
	Hexadecenoylcarnitine (C16:1)	Acylcarnitine	0.0249	1.316
	PC ae C38:3	Phosphatidylcholine	0.0383	1.144

q: FDR-adjusted *p* value; FC: fold change of the metabolites in progressor with respect to non-progressor; DMN, diabetic nephropathy; HTN, hypertensive nephropathy; PKD, polycystic kidney disease.

Table S2. DeLong's test for pairwise correlated ROC curves.

Group	Model 1 vs. Model 2	Model 1 vs. Model 3	Model 2 vs. Model 3
**DMN**	0.4258	0.0389	0.0300
**HTN**	0.0014	3.36E-05	0.0133
**PKD**	8.71E-04	3.54E-06	0.0195

Model 1, clinical only; Model 2, clinical + metabolites; Model 3, clinical + metabolites in random forest.
